# Biomimetic and bioinspired nano‐platforms for cancer vaccine development

**DOI:** 10.1002/EXP.20210263

**Published:** 2023-04-25

**Authors:** Chenchao Feng, Peng Tan, Guangjun Nie, Motao Zhu

**Affiliations:** ^1^ CAS Key Laboratory for Biomedical Effects of Nanomaterials & Nanosafety, CAS Center for Excellence in Nanoscience National Center for Nanoscience and Technology Beijing China; ^2^ Center of Materials Science and Optoelectronics Engineering University of Chinese Academy of Sciences Beijing China; ^3^ Klarman Cell Observatory Broad Institute of MIT and Harvard Cambridge USA; ^4^ GBA Research Innovation Institute for Nanotechnology Guangzhou China

**Keywords:** biomimetic and bioinspired nanomaterials, cancer immunotherapy, cancer vaccines, nanomedicine, targeted delivery

## Abstract

The advent of immunotherapy has revolutionized the treating modalities of cancer. Cancer vaccine, aiming to harness the host immune system to induce a tumor‐specific killing effect, holds great promises for its broad patient coverage, high safety, and combination potentials. Despite promising, the clinical translation of cancer vaccines faces obstacles including the lack of potency, limited options of tumor antigens and adjuvants, and immunosuppressive tumor microenvironment. Biomimetic and bioinspired nanotechnology provides new impetus for the designing concepts of cancer vaccines. Through mimicking the stealth coating, pathogen recognition pattern, tissue tropism of pathogen, and other irreplaceable properties from nature, biomimetic and bioinspired cancer vaccines could gain functions such as longstanding, targeting, self‐adjuvanting, and on‐demand cargo release. The specific behavior and endogenous molecules of each type of living entity (cell or microorganism) offer unique features to cancer vaccines to address specific needs for immunotherapy. In this review, the strategies inspired by eukaryotic cells, bacteria, and viruses will be overviewed for advancing cancer vaccine development. Our insights into the future cancer vaccine development will be shared at the end for expediting the clinical translation.

## Graphical abstract

Harnessing the immune system to attack the tumor with cancer vaccines is being intensively studied. Nowadays, biomimetic and bioinspired nanotechnology provides new impetus for the designing concepts of cancer vaccines. The specific behaviors and endogenous molecules of each type of living entity (eukaryotic cell or microorganism) offer unique features to address specific needs for cancer vaccines.

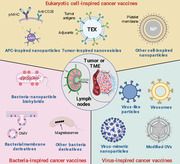

## 1 INTRODUCTION

Cancer, being a great threat to human lives, has never slowed down its pace.^[^ ^1^ ^]^ Over the past few decades, the paradigm of cancer treatment has undergone a major shift away from nonspecific chemo‐drugs to targeted and immune‐based approaches.^[^ ^2^ ^]^ Vaccine, a key player in fighting against infectious diseases in the past,^[^ ^3^ ^]^ has been applied to the treatment of cancer, trying to not only prevent^[^ ^4^ ^]^ but cure^[^ ^5^ ^]^ the disease. By training the host immune cells, cancer vaccines amplify the frequency and strength of pre‐existing immune responses or perhaps produce some de novo reactions, which can effectively eradicate local and disseminated metastatic tumors and establish long‐term immune memory to suppress tumor recurrence.^[^ ^6^ ^]^

**FIGURE 1:**
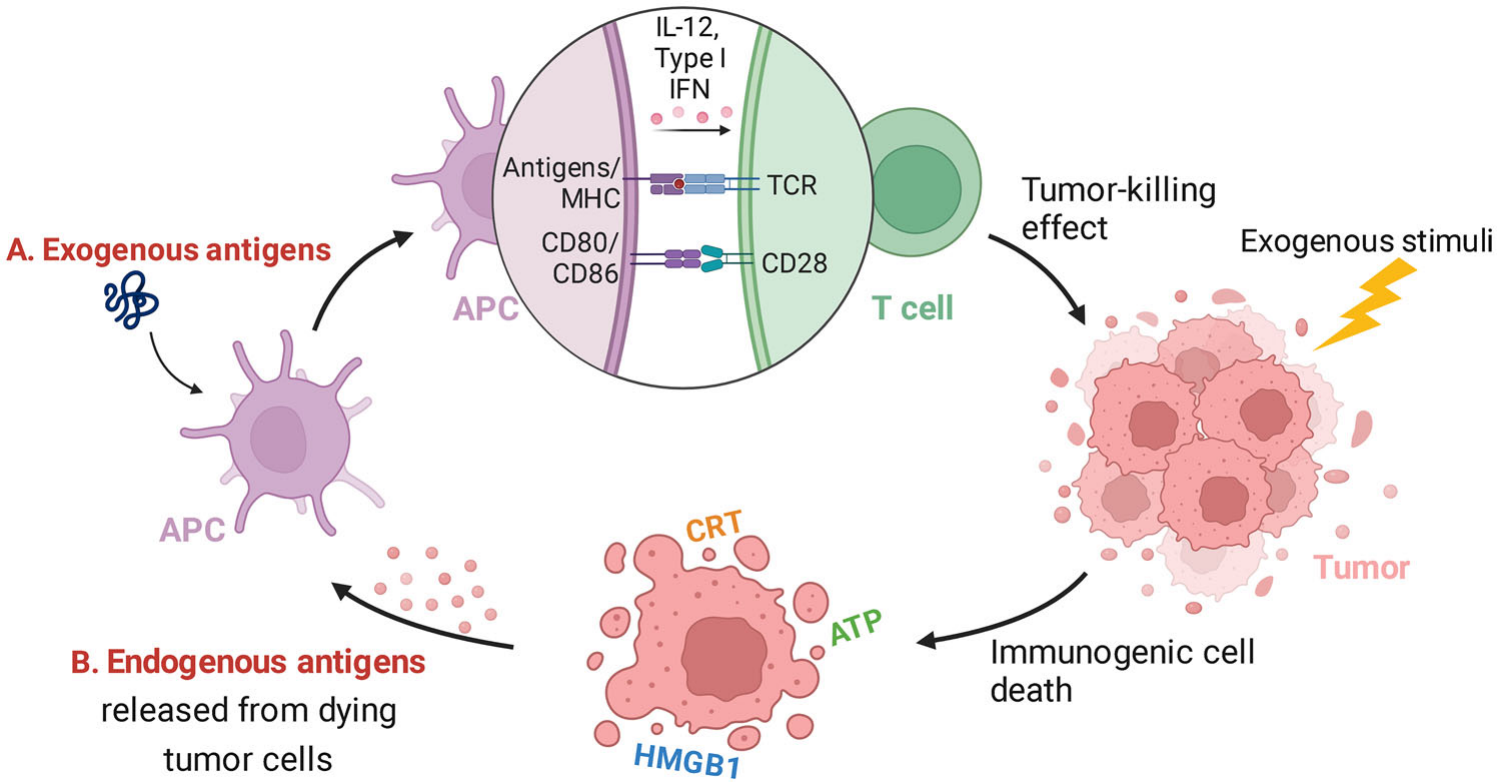
Schematic illustration of cancer vaccines based on exogenous or endogenous antigens. (A) Exogenous antigens are taken up and processed by antigen‐presenting cell (APCs) after injection. Antigen/major histocompatibility complex complexes can stimulate T cells together with costimulatory signals (such as CD80/CD86) and cytokines (such as interleukin‐12 and type I interferon). The activated T cells recognize tumor cells for specific killing. (B) Endogenous antigens and damage‐associated molecular pattern (DAMPs) (such as calreticuli, extracellular ATP, and high mobility group box protein 1) are released from tumor cells after they are exposed to exogenous stimuli and undergo immunogenic cell death. DAMPs act as danger signals and increase the immunogenicity of tumor cells, which promotes the uptake of endogenous antigens by APCs to elicit T cell response.

Typical cancer vaccines are designed for the delivery of tumor antigens to antigen‐presenting cells (APCs), particularly dendritic cells (DCs), for harnessing the power of host immunity against cancer. After taking up and processing the antigens, DC would present the immunogenic epitopes of antigens onto the major histocompatibility complex (MHC)‐I or MHC‐II molecules and migrate to the lymph nodes (LNs) for specific T cell recognition and activation. Notably, antigens are not necessarily delivered exogenously with the development of intrinsic cancer vaccination or in situ cancer vaccination.^[^ ^7^ ^]^ In situ cancer vaccination does not require the identification and isolation of the tumor antigens beforehand; on the contrary, it exploits tumor antigens available at the tumor site by promoting immunogenic cell death (ICD).^[^ ^7d^ ^]^ ICD, driven by pathogens, chemotherapeutics, physical cues, or necroptosis, can cause the release of antigens and, more importantly, damage‐associated molecular patterns, such as calreticulin (CRT), extracellular ATP, and high mobility group box protein 1 (HMGB1).^[^ ^7c^ ^]^ These factors, by binding to low‐density lipoprotein receptor‐related protein 1, P2X7, and toll‐like receptor 4 (TLR4), respectively, at the DC surface, enhance the functions of DC^[^ ^7a^ ^]^ to further exert the abscopal effect,^[^ ^7b^ ^]^ yielding a systemic immune response (Figure 1).

Numerous clinical studies of therapeutic cancer vaccines have implied the desirable properties associated with cancer rejection for vaccine design.^[^ ^5^ ^]^ After unremitting efforts, cancer vaccines ushered in a milestone in April 2010. Sipuleucel‐T (Provenge; Dendreon), an autologous DC‐based prostate cancer vaccine, became the first FDA‐approved human therapeutic cancer vaccine.^[^ ^8^ ^]^ Other vaccine formulations based on DNA,^[^ ^9^ ^]^ RNA,^[^ ^10^ ^]^ and synthetic long peptides^[^ ^11^ ^]^ have also demonstrated their efficacy in clinical trials. Promising though they seem to be, Sipuleucel‐T only improved the median survival by 4.1‐month (25.8 months in the Sipuleucel‐T group vs. 21.7 months in the placebo group) and no other therapeutic cancer vaccine has been approved over the past decade.^[^ ^8^ ^]^ The reasons for the moderate clinical outcomes of cancer vaccines are speculated: (i) the lack of suitable tumor antigens^[^ ^12^ ^]^ and optimized adjuvant components for eliciting a robust immune response against heterogenetic tumor cells;^[^ ^13^ ^]^ (ii) the soluble long‐peptides suffer from poor antigen presentation thus limiting the antigen‐specific CD8^+^ T cell recognition;^[^ ^14^ ^]^ (iii) the immunosuppressive tumor microenvironment (TME) attenuates the T cell activity for tumor killing.^[^ ^15^ ^]^

**FIGURE 2:**
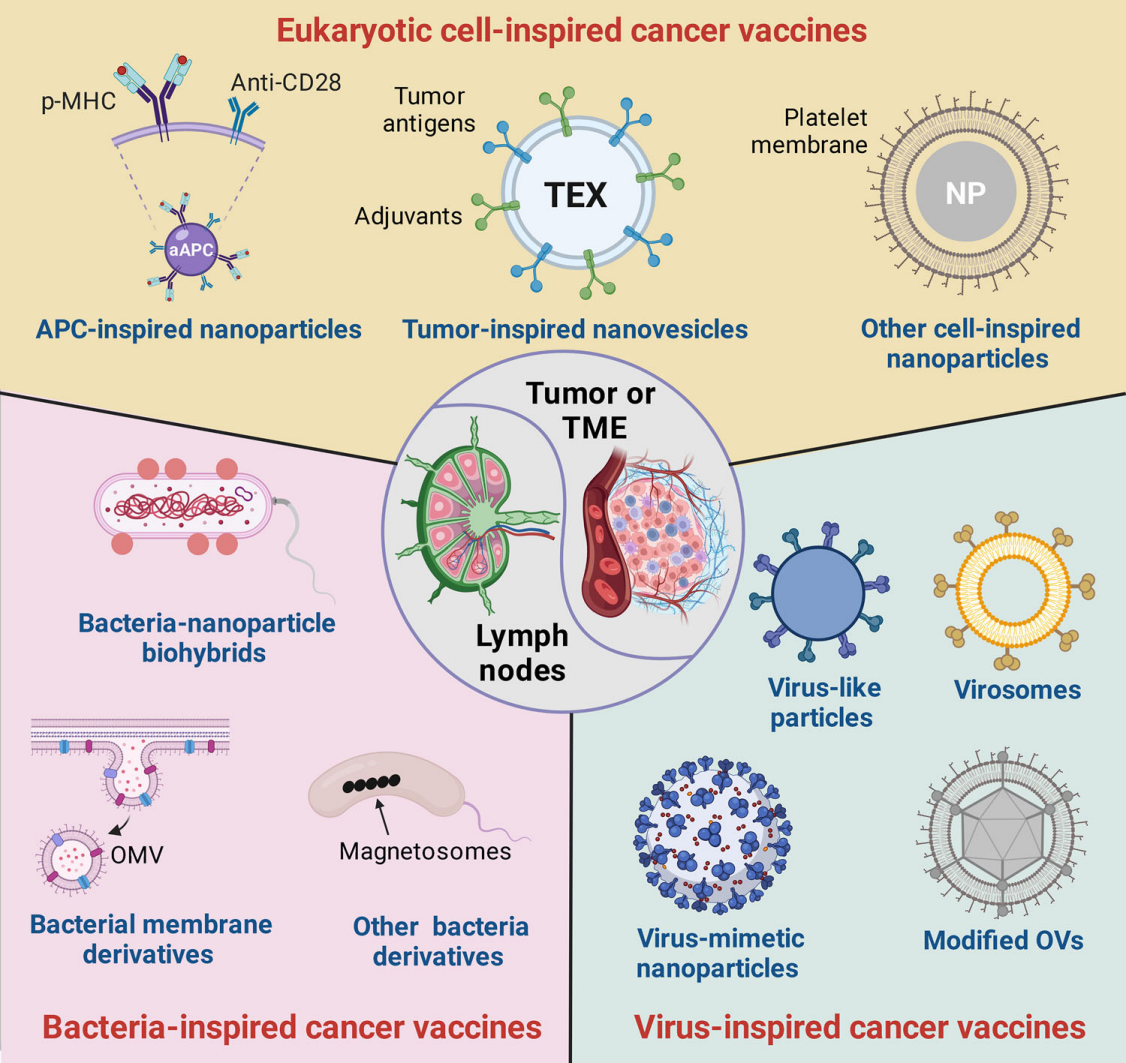
Schematic illustration of various biomimetic and bioinspired cancer vaccines. TME, tumor microenvironment; p‐MHC, peptide‐major histocompatibility complex; APC, antigen‐presenting cell; aAPC, artificial APC; TEX, tumor‐derived exosomes; NP, nanoparticle; OMV, outer membrane vesicle; OVs, oncolytic viruses.

Nanotechnology, which has been widely applied for drug delivery in cancer treatment,^[^ ^16^ ^]^ has shown its potential in vaccine delivery.^[^ ^14^, ^17^ ^]^ First, by fine tuning of physicochemical properties and the modification of targeting ligands, nanoparticles could accumulate in LNs and be efficiently taken up by APCs. Second, nanoparticles can escape from lysosomes through different mechanisms to facilitate exogenous antigens to be presented via MHC‐I pathway which is known as cross‐presentation for CD8^+^ T cell activation and proliferation.^[^ ^18^ ^]^ Meanwhile, some nanoparticles could directly deliver cargos into the cytoplasm through endocytosis‐independent pathways for an improved cross‐presentation efficiency. Functional motifs, such as peptide,^[^ ^19^ ^]^ DNA,^[^ ^20^ ^]^ and small molecules,^[^ ^21^ ^]^ could facilitate the fusion of lipid‐based nanoparticles with the plasma membrane for direct cytosolic delivery. A biomimetic fusogenic liposome prepared by fusing the conventional liposome with ultra‐violet inactivated Sendai virus was reported to directly deliver molecules into the cytoplasm and successfully induce antigen‐specific cytotoxic T lymphocytes (CTL) responses.^[^ ^22^ ^]^ Third, nanoparticles can encapsulate different types of antigens and adjuvants, so that the maximized immune response can be achieved by the co‐delivery of essential components to the same APC. Nanoparticles assembled by immunogenic materials may also be self‐adjuvanted.^[^ ^23^ ^]^ Lately, scientists endeavor to learn from nature and reproduce the complicated functions and interactions in living systems for vaccine development. A living entity (i.e. cell) has various endogenous substances and a cell‐type specific behavior that can guide us on the selection of biomaterials and “add on” functions for the design of efficient vaccines. Furthermore, the interactions between biomimetic and bioinspired nanocarriers and target cells greatly resemble the cell‐cell and pathogen (such as viruses and bacteria)‐cell interactions. For example, cell membrane coating confers desirable properties, such as long circulation time and specific targeting ability, to nanovaccines for parental cell‐like behaviors.^[^ ^24^ ^]^ Bacterial derivatives have been developed as self‐adjuvanted vaccine vectors by taking the advantage of their inherent immunostimulatory molecules.^[^ ^25^ ^]^ Virus‐like particles (VLPs) and virosomes have also made substantial progress in vaccine delivery owing to their natural tropism, the ability to cross biological barriers, as well as certain immunogenicity.^[^ ^26^ ^]^ In this review, we will focus on the biomimetic and bioinspired nanoscale delivery platforms for cancer vaccine development. We will describe the major strategies for biomimetic and bioinspired cancer vaccine design and summarize the cutting‐edge techniques, including various cancer vaccine formulations inspired by eukaryotic cells, bacteria, and viruses (Figure 2). To distinguish between biomimetic and bioinspired strategies in this review, “biomimetic” technique refers to the nature‐derived or semisynthetic delivery systems that are partly or entirely composed of biocomponents (i.e., exosomes and cell/bacterial membrane‐camouflaged nanoparticles). By contrast, “bioinspired” technique mainly refers to the synthetic systems that imitate the features of natural living entities [i.e., artificial APCs (aAPCs) and virus‐mimetic nanoparticles].^[^ ^27^ ^]^

## 2 TARGETING STRATEGIES FOR EXOGENOUS AND ENDOGENOUS CANCER VACCINES

**FIGURE 3:**
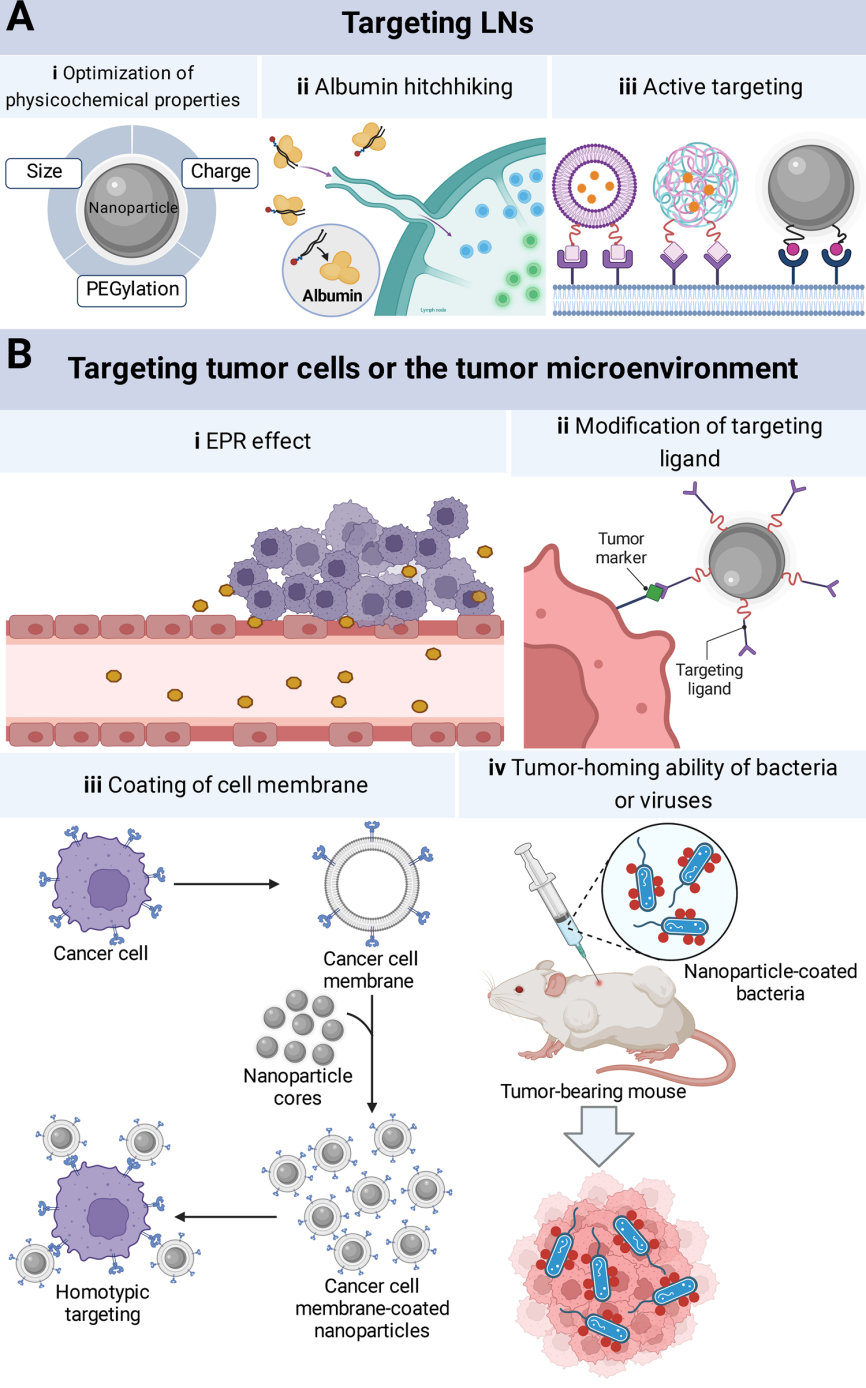
Targets and corresponding targeting strategies of cancer vaccines. (A) Lymph node (LN)‐targeting can be achieved by (i) optimization of nanoparticle physicochemical properties, such as size, charge, and PEGylation; (ii) albumin hitchhiking (cancer vaccine components bind to endogenous albumin for LN accumulation); and (iii) surface modification for active targeting; (B) tumor cell‐ or the tumor microenvironment‐targeting can be achieved by (i) enhanced permeability and retention effect; (ii) modification of the targeting ligands of proteins, carbohydrates, or lipids that overexpressed on tumor cells or within the TME; (iii) coating of cell membrane (i.e., cancer cell membrane) to gain the inherent homotypic binding ability; and (iv) harnessing the tumor‐homing ability of bacteria or viruses.

A potent immune response can be induced by (i) targeting the peripheral immune system, especially LNs, which are important sites for exogenous antigen presentation to T cells, and (ii) targeting tumor cells or the TME to elicit ICD or ameliorate immunosuppression (Figure 3).^[^ ^28^ ^]^ To treat tumor‐specific antigen (TSA)/tumor‐associated antigen (TAA) highly expressed tumors or highly mutated “hot” tumors, exogenous vaccines with LN‐targeting feature might be more efficient in inducing antigen‐specific T cell responses by providing an adequate number of exogenous antigens for immune education. By contrast, in low‐immunogenetic tumors such as TSA/TAA low‐expressed tumors or “cold” tumors, exogenous vaccines might be ineffective to induce sufficient T cell response against cancer. Endogenous strategies that induce ICD and the release of endogenous antigens are likely to trigger a more effective anti‐tumor immune response. By introducing the targeting ability of exogenous and endogenous cancer vaccines, we endeavor to elucidate the mechanism of action of these cancer vaccines by taking advantage of biomimetic and bioinspired materials.

### 2.1 Targeting the LNs

Various immune cell populations are present in the LNs, including CD4^+^ helper T cells, CD8^+^ T cells, B cells, and importantly, APCs. Targeted delivery of exogenous antigens to APCs is a primary task for cancer vaccine since the antigen presentation by APCs is the first step to initiate an intact adaptive immune response.^[^ ^29^ ^]^ Currently, there are three main strategies for nanovaccine LNs targeting: (i) tailoring physicochemical properties to permit passive transport of nanoparticles to LNs; (ii) modifying the nanovaccine with APC‐specific ligands or antibodies for active targeting;^[^ ^30^ ^]^ or (iii) hitchhiking on the endogenous molecule or cell.

It has been reported that the vaccine delivery systems with a size of around 10–100 nm can efficiently cross the interstitium and passively drain into LNs via lymphatic capillaries.^[^ ^29^ ^]^ Particles with a larger size (500–2000 nm) are mainly retained in the stroma where the transport to LNs relies on the cellular uptake.^[^ ^31^ ^]^ While modification of PEG has been shown to improve the stability of cancer vaccines and LN‐targeting, PEGylated nanoparticles may induce anti‐PEG immunoglobulin M antibodies, resulting in fast blood clearance.^[^ ^32^ ^]^ By contrast, taking advantage of the long circulation ability of red blood cells, erythrocyte membrane‐camouflaged nanoparticles could imitate RBCs and achieve long‐term circulation in comparison to PEG‐coating.^[^ ^33^ ^]^ Surface charge is another key factor that affects both LN‐targeting and APC uptake. Negatively charged nanoparticles are more likely to avoid being trapped in the interstitium for efficient LNs transportation while positively charged vehicles are more efficiently phagocytosed by DCs once reached the LNs.^[^ ^34^ ^]^ Thus, a robust immune response requires careful design to strike a balance between LNs targeting and APC uptake.

In addition to the passive transport strategies, conjugating specific ligand to nanoparticles could enable the active homing to the LNs through specific binding to APCs. C‐type lectin receptors are the most important class of APC‐specific targeting receptors which share primary structural homology in their carbohydrate‐recognition domain, including DC‐SIGN, mannose receptor, DEC‐205, and so on.^[^ ^35^ ^]^ Antibodies and multiple carbohydrate molecules such as mannose,^[^ ^36^ ^]^ galactose,^[^ ^37^ ^]^ dextran,^[^ ^38^ ^]^ and high‐mannose glycan^[^ ^39^ ^]^ have been developed for active targeting. The combination of cell labeling and bioorthogonal chemistry is another approach to actively delivering exogenous immunomodulatory agents to LNs. Metabolic glycoengineering of unnatural sugars^[^ ^40^ ^]^ and the insertion of lipid molecules into cell membranes^[^ ^41^ ^]^ can lead to the stable azido labeling of DCs and lymphatic endothelial cells, respectively. Besides, equipped with different pathogen associated molecular patterns (PAMPs), some pathogen derivatives, such as outer membrane vesicles (OMVs) can target LN or immune cells due to their “non‐self” characteristics.^[^ ^42^ ^]^

Owing to the natural lymphatic tropism of albumin,^[^ ^43^ ^]^ a strategy called “albumin hitchhike” has attracted a lot of attention by linking vaccine components to endogenous albumin for LN targeting. Antigens/adjuvants modified with a lipophilic albumin‐binding domain inclined to accumulate in LNs after injection, through in situ complexing with endogenous albumin. Compared to the traditional vaccines, the “albumin hitchhike” strategy triggered 30‐fold increases in T cell priming, resulting in sustained regression of TC‐1 tumors.^[^ ^44^ ^]^ This strategy was further applied to enhance the efficacy of CAR‐T therapy by attaching the ligand for a CAR to a polymer‐lipid tail (amph‐ligand). The CAR ligand‐conjugated lipid could bind to albumin to target LNs while inserting into the membrane of APCs for CAR‐T boosting. Amph‐ligand boosting elicited pronounced CAR‐T expansion and remarkable tumor reduction, thus leading to an extended survival in multiple solid tumor models. This approach could also be generalized to trigger the proliferation of CAR‐T toward any tumor target.^[^ ^45^ ^]^

### 2.2 Targeting tumor cells or the TME

Nanomedicine can be applied to exploit the ICD similarly to chemotherapeutic agents to prime immunity against a broad repertoire of tumor antigens.^[^ ^46^ ^]^ Furthermore, the combination of targeted delivery^[^ ^47^ ^]^ and stimuli‐responsive drug release^[^ ^48^ ^]^ can initiate immune response only at the tumor site. Due to the hyperpermeable tumor vasculature and inefficient lymphatic drainage from the tumor tissue, nanoparticles have a natural tendency to accumulate within tumors, which is called the “Enhanced Permeability and Retention (EPR) effect.”^[^ ^49^ ^]^ However, the EPR effect has been widely questioned for the lack of significant clinical translation and the biological differences between the mouse tumor models and the real human cancer pathology.^[^ ^50^ ^]^ Utilizing high‐affinity ligand modification onto the surface, NPs can be specifically retained at the tumor site by binding to the proteins, carbohydrates, or lipids that are overexpressed on tumor cells or within the TME.^[^ ^51^ ^]^ However, the efficiency of this active targeting is still far from satisfactory.^[^ ^51^ ^]^

An emerging targeting strategy is to camouflage nanoparticles with cell membranes, through which the cell surface proteins and receptors are preserved for nanoparticles to perform cell‐like functions, such as enhanced circulation, selective adherence to different disease substrates, and homotypic aggregation.^[^ ^27a^ ^]^ Therefore, various membrane‐camouflaged biomimetic nanoparticles for cancer‐targeted delivery have been developed.^[^ ^47^ ^]^ Owing to the overexpressed P‐selectin (a glycoprotein that can bind to CD44 receptors upregulated on the surface of cancer cells) on the platelet membrane, platelet membrane‐coated nanoparticles could specifically bind to CD44 receptor overexpressed on cancer cells and transport drug to the tumor site for apoptosis induction.^[^ ^52^ ^]^ Besides, cancer cell membrane coating represents a potent cancer‐targeting strategy by exploiting the inherent homotypic binding ability.^[^ ^53^ ^]^ In a recent study, the homologous targeting capability of patient‐derived cancer cell membrane‐camouflaged nanocarriers was confirmed in a preclinical setting, which demonstrated its feasibility in personalized cancer therapy.^[^ ^54^ ^]^

Apart from the eukaryotic cell membrane coating technology, bacteria‐facilitated delivery can achieve active tumor targeting through their intrinsic tumor‐homing ability. Certain bacteria strains have been shown to preferentially replicate within solid tumors when injected from a distal site owing to the immunosuppressive and unique TME,^[^ ^55^ ^]^ which makes bacteria an outstanding vector to deliver various therapeutic payloads to the tumor.^[^ ^56^ ^]^ An interesting example is attaching drug‐loaded nanoparticles to live bacteria and transport nanoparticles into tumors via the bacteria's tumor‐homing ability.^[^ ^57^ ^]^ Nano‐scaled bacterial‐derived components, such as bacterial ghosts (BGs), could retain some extent of the targeting properties of the parental bacteria for superior tumor‐targeted delivery.^[^ ^58^ ^]^

## 3 EUKARYOTIC CELL‐INSPIRED CANCER VACCINES

Cells in the body are communicating with each other all the time. Each cell type has its own unique characteristics, functions, and working modes. Naïve APCs are one of the most important cells for initiating active immune responses, while tumor cells display a rich pool of antigens. These cell‐type specific components and superior properties offer inspiration for scientists to tailor the cancer vaccines.

### 3.1 APC‐inspired cancer vaccine strategies

APCs, particularly DCs, are crucial for the initiation and regulation of innate and adaptive immune responses.^[^ ^59^ ^]^ Unfortunately, tumors have evolved with various escape mechanisms to evade immune recognition or redirect immune cells toward a dysfunctional, tolerogenic, or even immunosuppressive phenotype.^[^ ^60^ ^]^ Manipulating the behaviors and functions of APCs to reinforce the activation, expansion, and differentiation of T cells, is one of the key tasks of vaccination. Using bioinspired and biomimetic technology, APC‐mimicking nanoparticles or APC‐derived vesicles (i.e., exosomes) have been developed as novel vaccine platforms to activate T cells directly.

#### 3.1.1 aAPCs

By carefully decorating and tuning the presence of peptide‐MHC (p‐MHC) complexes and co‐stimulatory molecules on the surface, the aAPCs mimicked the natural APC interaction with T cells and promoted the T cell activation and expansion.^[^ ^61^ ^]^ aAPCs can be mainly classified into two categories: cell‐based systems and acellular systems.^[^ ^62^ ^]^ Cell‐based aAPCs are derived from human or xenogeneic cells, such as K562^[^ ^63^ ^]^ and mouse fibroblasts,^[^ ^61b^ ^]^ which are transfected with human leukocyte antigen (HLA), costimulatory signals, and other necessary molecules. Acellular aAPCs, ranging from synthetic microscale or nanoscale particles coated with functional antibodies or cell membranes to subcellular structures (exosomes), are more well‐defined and controllable compared with the cell‐based aAPCs.^[^ ^62^ ^]^

**FIGURE 4:**
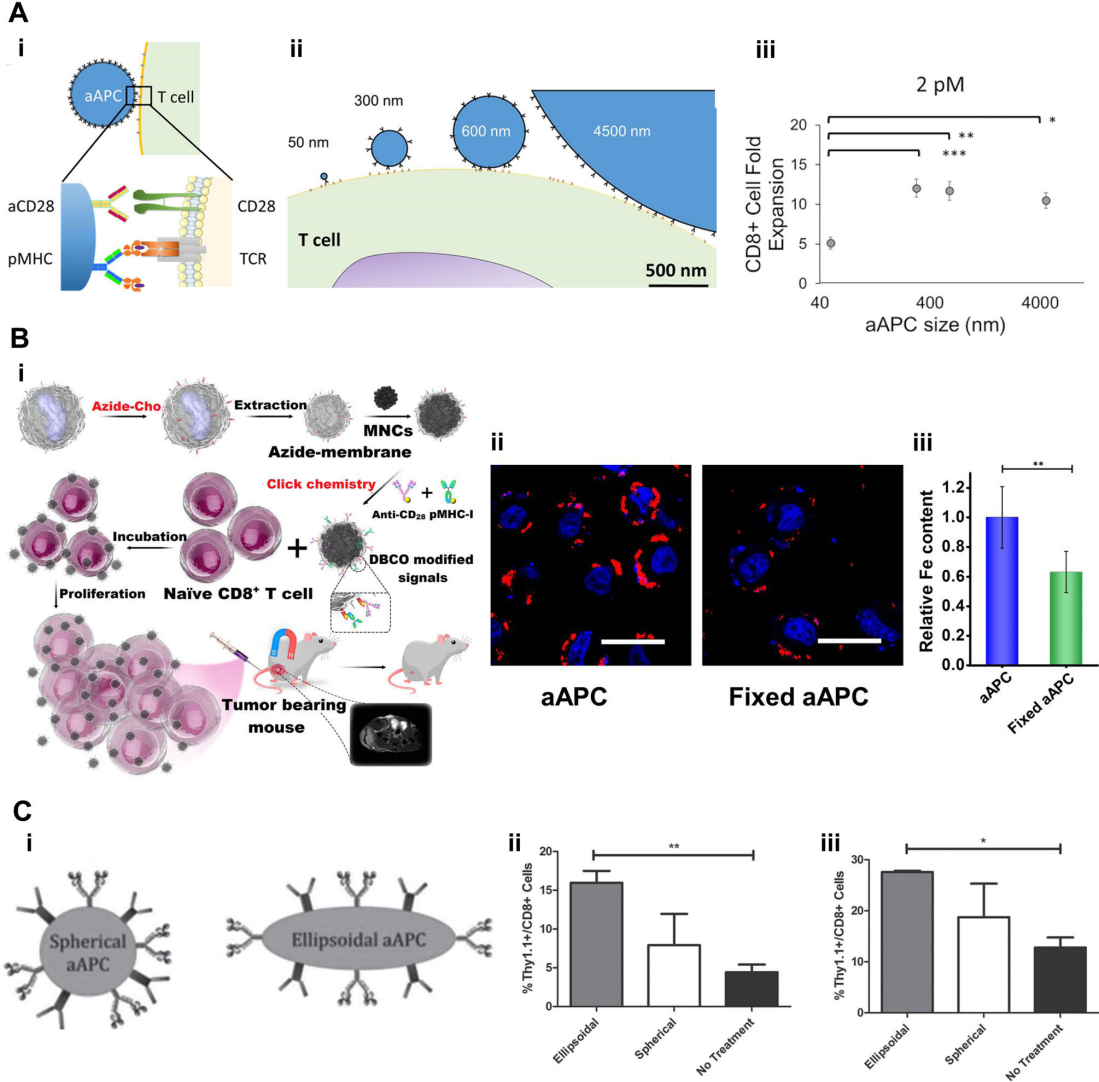
Different factors of artificial antigen‐presenting cells (aAPCs) affect their ability to activate CD8^+^ T cells. (A) The influence of particle size on aAPC‐triggered T cell expansion. (i) Schematic illustration of the interaction between nanoscale aAPC and antigen‐specific T cells. (ii) Schematic illustration of the impact of relative size and ligand density of aAPC on the interaction with T cell. (iii) Proliferation of CD8^+^ T cells induced by aAPCs of different sizes at a controlled total dose of 2 pM conjugated peptide‐MHC complexes. Reproduced with permission.^[^ ^64^ ^]^ Copyright 2017, American Chemical Society. (B) The impact of membrane fluidity of aAPC on T cell expansion. (i) Schematic illustration of the construction and application of leukocyte membrane‐coated aAPC. (ii) Fluorescence imaging of the interaction of T cells with aAPC/fixed aAPC. Red, aAPC; blue, nucleus. (iii) The relative iron content of aAPCs bound to CD8^+^ T cells based on ICP‐MS analysis of 1 × 10^6^ cells. The results (ii and iii) show that the interactions between aAPCs and T cells will be compromised once the membranes were fixed. Reproduced with permission.^[^ ^65^ ^]^ Copyright 2017, American Chemical Society. (C) The impact of particle shape of aAPC on T cell proliferation. (i) Schematic illustration of the ellipsoid and spheroid aAPC. The ellipsoid and spherical aAPCs along with 10^6^ antigen‐specific CD8^+^ T cells labeled Thy 1.1 were injected intravenously into irradiated mice. The “no treatment” group received T cells without aAPCs. The percentage of Thy 1.1^+^/CD8^+^ T cells in (ii) spleens and (iii) lymph nodes in mice on day 10 post injection. Reproduced with permission.^[^ ^66^ ^]^ Copyright 2015, John Wiley & Sons.

Many factors of nanoscale aAPCs have been studied for their contribution to vaccine efficacy, such as particle size, membrane fluidity, and shape. Hickey et al. synthesized a series of nanoscale aAPCs by conjugating p‐MHC complexes (signal 1) and anti‐CD28 antibody (signal 2) at a fixed molar ratio to nanoparticles of different sizes. It was found that small aAPCs (< 50 nm) boosted a 5‐fold T cell expansion compared to a 12‐fold expansion induced by large aAPCs (> 300 nm), demonstrating the size‐dependent effect (Figure 4A).^[^ ^64^ ^]^ Zhang et al. coated magnetic nanoclusters with azide‐modified leucocyte membranes and then conjugated them with stimulus signal via copper‐free click chemistry. The magnetic aAPCs not only facilitated the proliferation of cytotoxic T cells but also bound stably to T cells for magnetic‐guided tumor targeting and visualization under magnetic resonance imaging. An interesting phenomenon was found that the cell expansion was decreased when crosslinking the leucocyte membrane layer of aAPCs with glutaraldehyde, which indicated the importance of membrane fluidity (Figure 4B).^[^ ^65^ ^]^ Non‐spherical, anisotropic nanoscale aAPCs were synthesized for their ideal interfacial geometry due to the microscale radius of curvature for the long axis. Meyer et al. constructed biodegradable ellipsoid aAPCs which diminished non‐specific cell uptake and displayed excellent pharmacokinetic profiles over the spherical aAPCs. When intravenously injected in vivo, there was a notable increase in the proliferation of T cells mediated by ellipsoidal aAPC compared to either spherical aAPC‐treated or control groups (Figure 4C).^[^ ^66^ ^]^

#### 3.1.2 APC‐derived exosomes

Exosomes are saucer‐shaped membrane‐derived vesicles secreted by various cells, with a diameter of 30–100 nm.^[^ ^67^ ^]^ DC‐derived exosomes (DEX) harboring surface molecules from parental DCs, including MHC and costimulatory molecules, could maintain DC‐like immunostimulatory functionality.^[^ ^68^ ^]^ It was reported that exosomes derived from tumor peptide‐pulsed DCs could prime antigen‐specific CTLs and induce tumor growth suppression.^[^ ^69^ ^]^ However, the direct stimulation of T cells by DEX appears to be 10–20‐fold less efficient than the parental DCs.^[^ ^70^ ^]^ The immune activation capacity of DEX can be improved via the transferring of p‐MHC complexes to bystander APCs with or without reprocessing.^[^ ^68^, ^70^ ^]^ It was also reported that DEX could increase the immunogenicity of tumor cells via the transfer of HLA‐DR and CD86 molecules from DEX to the tumor cells.^[^ ^71^ ^]^

Owing to the superior properties, DEX appears to be a promising candidate for cancer vaccines.^[^ ^72^ ^]^ In a recent study, Fan et al. reported the anti‐CD3 and anti‐endothelial growth factor receptor (EGFR) antibody‐modified DEX for precise solid tumor therapy. The DEX maintained the immunostimulatory molecules of mature DCs, such as p‐MHC and CD86, for endogenous T cell activation, while the modification of αCD3 and αEGFR on DEX could connect the T cells and cancer cells by simultaneously binding to CD3 (expressed on T cell surface) and cancer cell‐associated EGFR. The bi‐specific DEX inhibited tumor recurrence and metastasis.^[^ ^73^ ^]^ Besides, macrophage‐derived exosome vaccine was reported to polarize M_2_ macrophages to M_1_ therapeutic phenotype.^[^ ^74^ ^]^

### 3.2 Tumor cell‐inspired cancer vaccine strategies

Typical cancer vaccines are formulated using tumor‐derived peptides or recombinant tumor antigenic proteins that are confined by HLA‐restriction. Given that, an alternative approach is to use tumor cells as an antigen source to directly induce a wide range of polyclonal tumor‐specific responses.^[^ ^75^ ^]^ A series of clinical trials have proved the efficacy of tumor cell‐based vaccines (Table 1).^[^ ^76^ ^]^

**TABLE 1:** Biomimetic and bioinspired cancer vaccines in preclinical or clinical trials.

Biomimetic/bioinspired approaches		Active pharmaceutical ingredient(s)	Indication(s)	Phase	ClinicalTrials.gov identifier (or refs.)
Eukaryotic cell‐inspired cancer vaccines	Artificial APCs (aAPCs)	MART1 peptide‐pulsed K562‐based aAPCs and interleukin (IL)−2/IL‐15	Melanoma (skin)	Phase I	NCT00512889
	APC‐derived exosomes	Tumor antigen‐pulsed dendritic cell‐derived exosomes (Dex)	Non‐small cell lung cancer	Phase II	NCT01159288
	Cancer cell membrane‐camouflaged nanovaccines	Membrane (derived from genetically engineered CD80‐expressing cancer cells)‐coated nanoparticles	B16‐OVA tumor	Preclinical	^[^ ^78^ ^]^
	Apoptotic whole tumor cells	GM‐CSF gene‐transduced autologous or allogeneic tumor cells (GVAX) and Ipilimumab	Prostate cancer	Phase I	NCT01510288
		Live‐attenuated listeria monocytogenes‐expressing mesothelin (CRS‐207), GVAX, and cyclophosphamide	Metastatic pancreatic cancer	Phase II	NCT01417000
	Tumor‐derived exosomes	Ascites‐derived exosomes and GM‐CSF	Colorectal cancer	Phase I	^[^ ^172^ ^]^
	Red blood cell (RBC)‐camouflaged nanovaccines	RBC membrane‐camouflaged PLGA nanoparticles loaded with antigen hgp100_25−33_ and monophosphoryl lipid A	Melanoma	Preclinical	^[^ ^96^ ^]^
	Platelet membrane‐ camouflaged nanovaccines	Platelet membrane‐camouflaged nanoparticles loaded with metformin and photosensitizer IR780	4T1 breast tumor	Preclinical	^[^ ^99^ ^]^
Bacteria‐inspired cancer vaccines	Bacteria‐nanoparticle biohybrids	Live attenuated *Salmonella* coated with DNA (encoding VEGFR2)‐loaded nanoparticles	B16 melanoma tumor	Preclinical	^[^ ^107^ ^]^
	Bacteria ghost (BG)	Cancer cell lysate‐loaded BGs together with interferon (IFN)‐α and GM‐CSF	Ex vivo	Preclinical	^[^ ^112^ ^]^
	Outer‐membrane vesicles (OMVs)	Escherichia coli OMVs loaded with antigens	B16‐OVA melanoma and colorectal cancer	Preclinical	^[^ ^42b^ ^]^
	Bacterial membrane‐coated nanovaccines	*Salmonella* OMV‐coated polymeric micelles (loaded with tegafur)	B16F10 melanoma	Preclinical	^[^ ^122^ ^]^
	Magnetosomes	Poly‐l‐lysine‐coated magnetosomes and an alternating magnetic field	U87‐Luc tumor	Preclinical	^[^ ^125^ ^]^
	Spores	*Clostridium* novyi‐NT spores	Solid tumor malignancies	Phase I	NCT01924689
		*Clostridium* novyi‐NT spores	Solid tumor malignancies	Phase I	NCT01118819
Virus‐inspired cancer vaccines	Virus‐like particles (VLPs)	Bacteriophage Qβ‐VLPs loaded with A‐type CpGs and Nivolumab	Melanoma	Phase II	NCT03618641
		A Melan‐A VLP vaccine alone or in combination with different adjuvants	Malignant melanoma	Phase II	NCT00651703
	Virosomes	Influenza virosomes including five melanoma epitopes	Melanoma	Phase I/II	^[^ ^173^ ^]^
	Virus‐mimetic nanoparticles	Virus‐mimetic nanoparticles loaded with melanoma‐associated gp100 epitope and CpG	B16‐F10 melanoma tumor	Preclinical	^[^ ^152^ ^]^
	Oncolytic virus	An oncolytic vaccinia virus Pexa‐Vec, Durvalumab, and Tremelimumab	Colorectal cancer	Phase I/II	NCT03206073
		A replication‐competent herpes simplex virus−1 oncolytic virus HF10 and Ipilimumab	Malignant melanoma	Phase II	NCT02272855

Abbreviations: APC, antigen‐presenting cell; MHC, major histocompatibility complex; OVA, ovalbumin; GM‐CSF, granulocyte‐macrophage colony‐stimulating factor; VEGFR2, vascular endothelial growth factor receptor 2.

#### 3.2.1 Cancer cell membrane‐camouflaged nanovaccines

Apart from the intercellular homologous binding ability mentioned above, the display of membrane‐attached tumor antigens made cancer cell membrane a potent candidate for antigen resource.^[^ ^77^ ^]^ Fang et al. first attempted to coat PLGA nanoparticles with membrane from B16‐F10 mouse melanoma cells via coextrusion, followed by incorporating with an adjuvant monophosphoryl lipid A (MPLA). These nanoparticles were shown to efficiently induce DC maturation for the subsequent activation of antigen‐specific T cell response.^[^ ^53^ ^]^

Owing to the global expression of MHC‐I on all types of cells including cancer cells, Jiang et al. transformed cancer cell membrane‐coated nanoparticle into a novel aAPC to directly stimulate T cells in the absence of professional APCs. Nanoparticles were coated with cell membrane originating from genetically engineered CD80‐expressing cancer cells. The biomimetic nanoparticle could bypass the step of traditional APC‐mediated antigen processing and directly activate antigen‐specific T cells through the engagement of the cognate T cell receptor and CD28, therefore successfully inhibiting tumor growth in both prophylactic and therapeutic tumor models.^[^ ^78^ ^]^

#### 3.2.2 Apoptotic tumor cells

Another common form of tumor cell‐based vaccines is apoptotic whole tumor cells which could be prepared by applying a lethal dose of ultraviolet ray irradiation to tumor cells.^[^ ^79^ ^]^ Early studies using irradiated whole tumor cells showed limited efficacy.^[^ ^80^ ^]^ Second‐generation apoptotic tumor cell vaccines using cytokine‐, chemokine‐ or costimulatory molecule‐transgenic tumor cells have emerged in preclinical and clinical trials (Table 1).^[^ ^80^ ^]^ Irradiated tumor cells that were genetically engineered to secrete granulocyte‐macrophage colony‐stimulating factor (GM‐CSF) were found to induce the most potent, long‐lasting, and specific anti‐tumor immunity compared to the other 9 immunomodulator‐transgenic cells in a B16 melanoma model.^[^ ^81^ ^]^ This GM‐CSF gene‐transduced autologous or allogeneic tumor cells, which was called GVAX, recruited APCs and boosted the uptake by APCs owing to the adjuvant effect of paracrine GM‐CSF secretion.^[^ ^82^ ^]^ Extensive preclinical data have supported the antitumor efficacy of GVAX, especially in the combination with other treatments, such as cytotoxic T‐lymphocyte protein 4 blocking antibody.^[^ ^83^ ^]^

Distinct from the genetical expression of GM‐CSF adjuvant, Fan et al. tethered CpG adjuvant‐loaded nanodepots onto the surface of apoptotic whole tumor cells. After being treated with a potent ICD‐inducing agent mitoxantrone, tumor cells underwent ICD and served as antigen sources. The nanodepots were constructed via the charge‐mediated complexation between cationic lipid vesicles containing a maleimide‐modified lipid and anionic thiolated hyaluronic acid, followed by chemical cross‐link‐mediated stabilization. The maleimide‐displaying CpG‐NPs were then attached to the surface of dying tumor cells that were pretreated with TCEP (a reducing agent to increase free sulfhydryl groups on the cell membrane). The loading of this CpG nanodepot significantly increased the survival rate of mice from 20% to 100%, compared to the unloaded counterparts, in a prophylactic B16F10‐Ovalbumin (OVA) tumor model. When combined with programmed cell death protein 1 antibody blockade therapy, this whole‐cell vaccine could completely eliminate the tumors in 78% of CT26 cancer‐bearing mice.^[^ ^84^ ^]^

#### 3.2.3 Tumor‐derived exosomes

Previous work demonstrated that tumor‐derived exosomes (TEX) harbor both neoantigens and TAAs.^[^ ^85^ ^]^ However, solely immunizing with TEX merely induced satisfied antitumor immunity due to the limited immunogenicity and immunosuppressive TME.^[^ ^86^ ^]^ Several approaches, such as genetic engineering, physical embedding, and surface protein conjugating, have been employed to co‐deliver multiple adjuvants or immunomodulators to improve the efficacy.

Morishita et al. constructed SAV‐LA‐expressing exosomes (SAV‐exo) via transfecting a plasmid encoding a fusion protein of streptavidin (SAV)‐lactadherin (LA; an exosomal surface protein) into tumor cells. A TLR agonist CpG ODN modified with biotin was linked to SAV‐exo by SAV‐biotin interaction. In a B16BL6 tumor model, the co‐delivery system induced a stronger antitumor effect than the simple mixture of exosomes and CpG.^[^ ^87^ ^]^ In another study, SAV‐exo was incorporated with biotinylated GALA (a pH‐sensitive fusogenic peptide that induces pore formation at pH 5) to achieve efficient cytosolic delivery and cross‐presentation of exosomal tumor antigens.^[^ ^88^ ^]^ Harvesting exosomes from dying tumor cells that underwent ICD is another approach to improving immunogenicity. Zhou et al. demonstrated that exosomes collected from the immunogenic dying tumor cells showed a higher level of CRT and HMGB than the ones isolated from normal tumor cells. To further block the C‐C chemokine receptor type 4/C‐C motif chemokine 22 axis which is vital for the recruitment of regulatory T cells, CCL22 siRNA was electroporated into exosomes. An immunogenic peptide MART‐1 (sequence: ELAGIGILTV) was further modified to the surface of exosomes for expanding specific CD8^+^ T cells after adoptive T cell transfer. The exosome‐based vaccines generated dual effects on both eliciting antitumor T cell responses and modulating the immunosuppressive TME for delayed tumor growth.^[^ ^89^ ^]^ Furthermore, radiation therapy could lead to the accumulation of cytosolic double‐stranded DNA (dsDNA) in cancer cells. dsDNA‐containing exosomes secreted by irradiated cancer cells have been shown to inhibit tumor growth in mice by activating DCs through a STING‐dependent pathway.^[^ ^90^ ^]^

TEX may also serve as antigen sources for DC vaccination. The DC loading strategy helps to overcome the risk of eliciting immunosuppressive features and inadequate induction of immune responses by TEX vaccine.^[^ ^86^ ^]^ In an earlier study, Wolfers et al. found that after the uptake of TEX, DCs induced potent CD8^+^ T cell‐dependent antitumor effects on syngeneic and allogeneic established mouse tumors, while free TEX failed to activate CTL.^[^ ^91^ ^]^ Similarly, Rao et al. indicated that DC pulsed with human HepG2 cell‐derived exosomes (DC_TEX_) could induce tumor rejection in both ectopic and orthotopic HCC mouse models. The DC_TEX_ treatment promoted the recruitment of effector T lymphocytes to tumor sites while reducing the number of immunosuppressive Treg cells. The reversal of TME could also be demonstrated by the elevated level of interferon (IFN)‐γ and decreased level of interleukin−10 and transforming growth factor‐β.^[^ ^92^ ^]^

### 3.3 Cancer vaccine strategies inspired by other types of cells

#### 3.3.1 Red blood cells

Due to the remarkable biocompatibility, biodegradability, long life‐span, and favorable encapsulation ability, red blood cells (RBCs) receive significant attention for drug delivery.^[^ ^93^ ^]^OVA‐entrapped RBCs were injected intravenously along with poly (I:C) into mice and induced the activation of OVA‐specific CD4^+^ and CD8^+^ T cells.^[^ ^94^ ^]^ An alternative approach is to utilize the entire RBC membrane as a coating material for long circulating vaccine generation.^[^ ^95^ ^]^ Guo et al. designed an RBC membrane‐camouflaged PLGA nanoparticles to co‐deliver antigen hgp100_25−33_ and adjuvant MPLA. The peptide was bound to PLGA via a disulfide bond to permit the release of antigens in the reductive intracellular milieu of DCs. Mannose was incorporated into RBC membranes via lipid insertion for actively targeting to DCs. The nanovaccine exhibited strong suppression of tumor growth in prophylactic, therapeutic, and metastatic melanoma models.^[^ ^96^ ^]^

#### 3.3.2 Platelets

Platelets, small anucleate cellular fragments released by megakaryocytes, are related to hemostasis, tumor metastasis, and other physiological and pathophysiological processes. Equipped with multiple “self‐recognized” proteins (such as CD47), the platelet membrane was reported to significantly inhibit phagocytosis‐mediated bloodstream clearance and particle‐induced complement activation, leading to a prolonged plasma half‐life of nanoparticles.^[^ ^52^, ^97^ ^]^ The long‐circulating properties, together with the tumor‐targeting ability (mentioned in Section 2.2), make platelet plasma membrane‐coated nanoparticle an ideal platform to transport cargos to tumor sites and act as an in situ cancer vaccine. Bahmani et al. constructed a platelet membrane‐cloaked nanoparticle for the intratumoral delivery of a TLR7/8 agonist R848, which prolonged retention at the tumor site and improved the interactions between NPs and various cells in the TME. Even administered at a low total dosage of 18 μg vaccine per mouse, 87.5% of vaccine‐treated mice eradicated tumors in an MC38 colorectal tumor model.^[^ ^98^ ^]^ Mai et al. developed an in situ vaccine by encapsulating metformin and photosensitizer IR780 within platelet membranes to achieve a long half‐life and significant accumulation in tumors. Metformin was able to inhibit the mitochondrial respiratory chain and reduce the O_2_ consumption of the tumor, which synergized with IR780‐induced ICD. Interestingly, the introduction of metformin also contributed to the reversal of the immunosuppressive microenvironment via reducing the infiltration of myeloid‐derived suppressor cells and Tregs. As a result, the multifunctional nanovaccine displayed a remarkable antitumor efficacy in vivo.^[^ ^99^ ^]^

Additionally, cell membrane‐coated nanovaccines derived from neutrophils,^[^ ^100^ ^]^ natural killer cells,^[^ ^101^ ^]^ and other eukaryotic cells have demonstrated their potential in cancer immunotherapy. The innovative top‐down strategy bestows parent cell‐mimicking properties while bypassing challenges that may be encountered in the bottom‐up manufacturing procedure. A variety of cell types can be chosen as membrane resources depending on the desired functions (Table 2).

**TABLE 2:** The main functions of various cell membrane‐camouflaged nanoparticles.

Types of cell membranes	Major functional molecule(s)	Function(s)	Refs
Cancer cell membrane	Tumor antigens Surface adhesion molecules	Antigen display Homotypic targeting	[53]
Red blood cell membrane	CD47	Long blood circulation	[174]
Platelet membrane	CD47 P‐selectin	Long blood circulation Selective binding to tumor cells, injured vasculature, and pathogen	[97] [52, 97]
Neutrophil membrane	Adhesion molecules (such as L‐selectin, LFA‐1, β1 integrin, and CXCR4)	Circulating tumor cells‐ and niche‐targeting	[100]
Natural killer cell membrane	Tumor‐targeting proteins (such as DNAM‐1 and NKG2D) NKCMs proteins (such as IRGM1, CB1, galectin‐12, RAB‐10, and RANKL)	Tumor targeting Inducing or enhancing the polarization of M1 macrophages	[101]

Abbreviations: LFA‐1, lymphocyte function‐associated antigen‐1; CXCR4, C‐X‐C chemokine receptor type 4‐A; DNAM‐1, DNAX accessory molecule 1; NKG2D, natural killer group 2D; NKCMs, natural killer cell membranes; IRGM1, immunity‐related GTPase family M member 1; CB1, cannabinoid receptor 1; RAB‐10, ras‐related protein rab‐10; RANKL, receptor activator of nuclear factor‐kappa B ligand.

## 4 BACTERIA‐INSPIRED CANCER VACCINES

Since the discovery of “Coley's toxins,”^[^ ^102^ ^]^ bacteria attracts increasing attention in biomedical applications due to their unique biological behaviors, such as tumor targeting, PAMP‐facilitated APC recognition, intratumoral penetration, native bacterial cytotoxicity, and controllable transcription by external signals.^[^ ^103^ ^]^ Apart from the roles of bacteria as therapeutic agents and tools of gene cloning, multiple nanoscale bacterial derivatives, such as BG, OMVs, endospores, as well as magnetosomes have proved their potential in drug delivery. Nanoparticle‐carrying bacteria termed “microbots” have also been reported, in which the cargo was not entrapped in the bacteria, but rather conjugated on the bacteria's surface.^[^ ^104^ ^]^ Herein, we will focus on the use of bacteria‐nanoparticle biohybrids and nanoscale bacterial derivatives for cancer vaccine development.

### 4.1 Bacteria‐nanoparticle biohybrids

The concept of bacteria‐nanoparticle biohybrid is to integrate bacteria with abiotic systems such as micro/nanoparticles to achieve synergistic or complementary effects.^[^ ^57^ ^]^ Since some bacteria, such as *Bifidobacterium*, *Clostridium*, and *Salmonella* have been found to preferentially replicate within solid tumors when injected from a distal site,^[^ ^105^ ^]^ hybridization of nanoparticles with the above bacteria can direct the nanoparticles to the tumor site and penetrate deeply into tumor tissue due to the bacteria motility. Besides, biohybrids can serve as a highly flexible platform for various treatment combinations by loading different types of therapeutic agents.^[^ ^106^ ^]^

**FIGURE 5:**
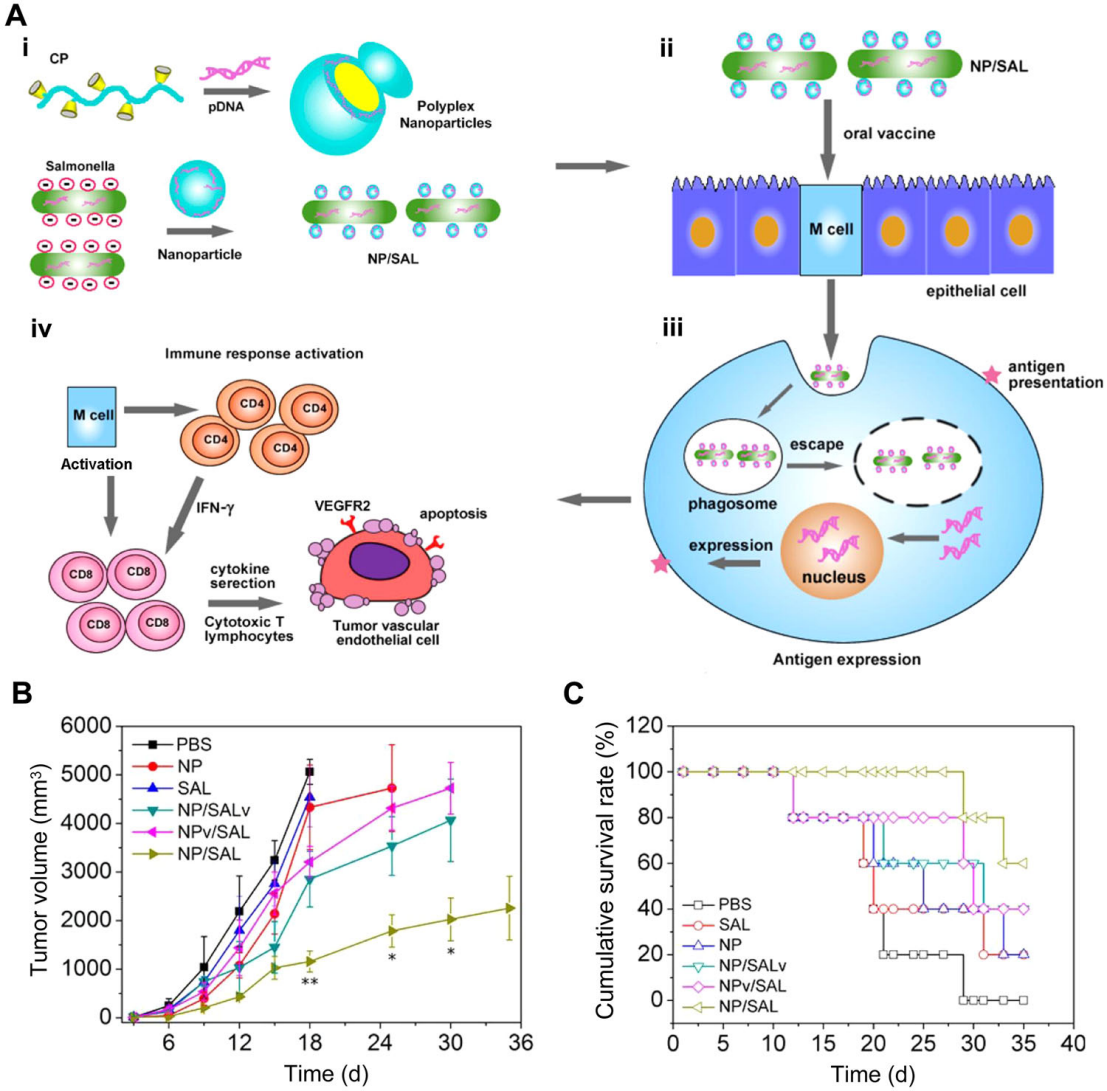
Nanoparticle‐coated bacteria as oral DNA vaccines to improve the antigen expression and immune activation. (A) Schematic illustration of the design and application of cationic nanoparticles‐coated attenuated *Salmonella* (NP/SAL). (i) Self‐assembly of polyplex nanoparticles from cross‐linked β‐cyclodextrin‐PEI600 (CP) and DNA plasmid (pDNA) and the attachment of the polyplex nanoparticles onto the live attenuated *Salmonella* surface. (ii) Delivery of oral DNA vaccine mediated by NP/SAL. (iii) Phagosome escape and antigen expression promoted by NP/SAL. (iv) Immune response elicited by biohybrid vaccine. (B) Mean tumor growth curve in a B16 melanoma model treated with different formulations. (C) Survival curves of tumor‐bearing mice treated with different formulations. NP, CP/pcDNA3.1‐VEGFR2; SAL, *Salmonella*‐pcDNA3.1‐VEGFR2; NP/SALv, biohybrid of CP/pcDNA3.1‐VEGFR2 complexes attaching on *Salmonellae* loaded with pcDNA 3.1 (empty vector); NPv/SAL, biohybrid of CP/pcDNA3.1 complexes attaching on *Salmonellae* loaded with pcDNA3.1‐VEGFR2; NP/SAL, biohybrid of CP/pcDNA3.1‐VEGFR2 complexes attaching on *Salmonellae* loaded with pcDNA 3.1‐VEGFR2. Reproduced with permission.^[^ ^107^ ^]^ Copyright 2015, American Chemical Society.

Hu et al. reported an oral DNA vaccine based on cationic nanoparticle‐coated bacterial vectors. Cross‐linked β‐cyclodextrin‐PEI600 nanoparticles loaded with DNA plasmid encoding autologous vascular endothelial growth factor receptor 2 (VEGFR2, a receptor overexpressed on tumor vasculatures) could assemble onto live attenuated *Salmonella* surface via electrostatic interaction. The nanoparticle coating offered a strong buffering capacity and large contact angle to protect *Salmonella* from the harsh acid environment in the stomach and boosted the dissemination of the bacteria into the blood. Meanwhile, the coated cationic nanoparticles ruptured the phagosomes after internalization through the “proton‐sponge” effect thus enhancing the VEGFR2 gene expression. Due to the T cell‐mediated inhibition of angiogenesis, the suppression of tumor growth was achieved by the biohybrid vaccination. The biohybrid treatment was at least 4 times and 3.7 times more potent than naked *Salmonellae* or nanoparticles, indicating a synergistic effect (Figure 5).^[^ ^107^ ^]^ The bacteria‐nanoparticle biohybrids are handy and simple to implement without the need for genetic manipulation. However, a balance of risks and benefits should be considered when using live bacteria for clinical translation.^[^ ^108^ ^]^

### 4.2 Bacterial membrane derivatives

#### 4.2.1 BG

BG is an empty envelope of Gram‐negative bacteria and is rid of the undesirable toxicity of living bacteria. BG is produced by the expression of lysis gene E which controls the formation of transmembrane tunnels in the bacterial membrane and the discharge of cytoplasmic content.^[^ ^109^ ^]^ Jalava et al. have elucidated the time course and formation process of BG in detail.^[^ ^110^ ^]^ As a novel vaccine delivery system, BG exhibits excellent natural adjuvant properties by possessing multiple PAMPs, such as LPS, flagellin, or peptidoglycan. Groza et al. reported that the application of BGs could enhance oxaliplatin‐mediated ICD and lead to an intense synergistic anticancer activity against the CT26 allograft, demonstrating the immune‐stimulatory potential of BGs.^[^ ^109b^ ^]^ Besides, BG has a high loading capacity by depositing drugs into different cellular compartments, including outer membrane, periplasmic space, inner membrane, and cytoplasmic space. Several techniques have also been developed for plugging the E‐lysis tunnel of BG to entrap antigens in the cytoplasmic space.^[^ ^111^ ^]^ Michalek et al. showed that cancer cell lysate‐loaded BGs enhanced DC maturation in the presence of IFN‐α and GM‐CSF, which could boost the proliferation of both autologous CD4^+^ and CD8^+^ T cells. The one‐step antigen delivery and DC maturation‐inducing BG‐based platform displayed a promising tool for the development of next‐generation clinical‐grade therapeutic cancer vaccines.^[^ ^112^ ^]^

#### 4.2.2 OMVs

OMVs, derived from the outer membranes of Gram‐negative bacteria, are spherical particles with a diameter of 20–250 nm.^[^ ^113^ ^]^ With various pathogen‐associated antigens and PAMPs inherited from the parental bacterium, OMVs have been used in prophylactic vaccination against infectious diseases induced by the corresponding pathogens.^[^ ^114^ ^]^ In the field of tumor immunotherapy, researchers found that OMV‐based formulations induced long‐term antitumor immune responses mediated by IFN‐γ.^[^ ^115^ ^]^ However, concerns were raised regarding the safety of OMVs, especially the potential inflammation, due to the enrichment of PAMPs.^[^ ^116^ ^]^ Park et al. adopted an unconventional isolation approach to producing artificial OMVs by using lysozyme and high pH treatment, resulting in pure vesicles with decreased cytosolic components including proteins, RNA and DNA. The artificial OMVs caused limited side effects even at a much higher dose than regular OMVs while exhibiting greater immunoadjuvant activity compared to other traditional adjuvants.^[^ ^117^ ^]^ Recently, Qing et al. reported an avenue to avoid the high toxicity and antibody‐dependent clearance of OMVs by shielding OMVs in a pH‐sensitive nanoshell comprising highly biocompatible calcium phosphate (CaP). The CaP shells prolonged the half‐life of circulation and greatly reduced the toxicity of OMVs. The slightly acidic pH of TME promoted the destruction of CaP shells which not only helped to modulate the acidic TME but also boosted the release of OMVs, leading to a significant immune response.^[^ ^118^ ^]^

Loading foreign antigenic peptides onto/into OMVs is a vital step to produce OMV‐based cancer vaccines. Besides the methods of genetic engineering or electroporation,^[^ ^119^ ^]^ Cheng et al. described a Plug‐and‐Display approach to fabricating a flexible tumor vaccine platform. Tumor antigens were conjugated on the surface of OMVs via fusion with ClyA protein, a common membrane protein on OMVs. Then the platform was optimized through the protein Plug‐and‐Display system, a SpyTag (SpT)/SpyCatcher (SpC) pair and a SnoopTag (SnT)/SnoopCatcher (SnC) pair. The SpC and SnC catchers were fused with ClyA on the OMVs surface so that the SpT‐ or SnT‐labeled antigens could be linked to OMVs readily via isopeptide bonds between the tags and catchers. The bioengineering approach allowed the platform to display multiple tumor antigens flexibly and exert antigen‐specific T cell‐mediated anticancer responses.^[^ ^42b^ ^]^ Alternatively, cancer antigens can be loaded by fusing cancer cell membrane with bacteria OMVs to generate hybrid vesicles that simultaneously retained both antigens and natural adjuvant components.^[^ ^120^ ^]^

#### 4.2.3 Bacterial membrane‐camouflaged nanoparticles

In addition to natural bacterial membrane derivatives, bacterial membrane‐camouflaged nanoparticles have been explored. Nanoparticles loaded with therapeutic cargos can synergize with the immunostimulatory molecules of bacteria membranes.

In one example, OMV‐B16 cancer cell (CC) membrane hybrid vesicles were coated onto hollow polydopamine (HPDA) NPs (HPDA@[OMV‐CC]). The homing ability of the CC membrane components allowed HPDA@[OMV‐CC] to specifically accumulate in tumor sites. Under NIR irradiation, HPDA NPs generated a significant photothermal effect in vivo and fully eradicated melanoma together with immunostimulatory components of OMV, without notable adverse effects.^[^ ^121^ ^]^ In a second study by Chen et al., *Salmonella* OMVs modified with non‐fouling PEG and targeting ligand RGD peptide were coated on the tegafur [a prodrug of fluorouracil (5‐FU)]‐loaded polymeric micelles. Tegafur triggered tumor cell apoptosis due to the chemotherapeutic effect, which was speculated to exert a “vaccine‐like effect” on the release of tumor antigens. Furthermore, 5‐FU could sensitize cancer cells for CTL recognition and specific killing. The combination of bacterial‐ and chemo‐therapeutics generated an effective protective immunity and inhibited tumor metastasis to the lungs.^[^ ^122^ ^]^

### 4.3 Other nanoscale bacteria derivatives

Magnetotactic bacteria are capable to respond to external magnetic fields due to their unique organelles, called magnetosomes, where nanoscale crystals of magnetic iron minerals are stored.^[^ ^123^ ^]^ The most important application of magnetosomes in cancer therapy is magnetic hyperthermia. Alphandéry et al. demonstrated that magnetosomes have a high absorption rate and a more homogeneous temperature distribution. Meanwhile, magnetosomes could be internalized by cancer cells or bind the cytomembranes under a magnetic field. Therefore, magnetosome‐based thermotherapy is more efficient compared with other materials including two different types of synthesized superparamagnetic iron oxide nanoparticles.^[^ ^124^ ^]^ In a subsequent study, the researchers removed most endotoxins and organic pyrogenic materials from magnetosomes for safety and used poly‐l‐lysine as a coating material. All the mice bearing intracranial U87‐Luc tumors were completely cured after the intratumoral administration of magnetosomes followed by magnetic sessions. Multiple mechanisms besides hyperthermia, such as tumor apoptosis and polynuclear neutrophil recruitment, may contribute to the antitumor response, indicating the potential of magnetosomes in combination with immunotherapy.^[^ ^125^ ^]^ Nevertheless, some researchers recently proposed that magnetosomes were more efficient and viable for photothermal therapy since photothermia was 100 to 1000 times‐fold more efficient than magnetic hyperthermia after the cellular internalization.^[^ ^126^ ^]^

Finally, spores, which are produced by bacteria expressing sporulation‐specific genes and originally serve as a preservation mechanism against the adverse external environment, have been used in cancer therapy owing to their property of preferential replication in hypoxic tumor regions.^[^ ^127^ ^]^ In a recent phase I clinical study, 24 patients with injectable, treatment‐refractory solid tumors received a single intratumoral injection of *Clostridium* novyi‐NT spores. 41% of the patients showed a reduced tumor volume and half of the tumors with spores germination had elevated infiltration of T cells and myeloid cells, indicating the immunostimulatory capacity of C. novyi‐NT spores.^[^ ^128^ ^]^ The combination of spores with other therapies, such as cancer chemotherapy^[^ ^129^ ^]^ and radiation therapy,^[^ ^130^ ^]^ has also been explored.

## 5 VIRUS‐INSPIRED CANCER VACCINES

With a better understanding of the virus's working mechanism, it becomes practical to transform the virus from a pathogen to a fine‐tuned multifunctional delivery platform. Viruses are vehicles that efficiently transfer their genes into hosts for self‐replication. The unique properties, including shapes, well‐defined surface elements, and rigorously ordered structure, provide clues for biomimetic and bioinspired delivery system design.^[^ ^131^ ^]^ VLPs, virosomes, and virus‐mimetic nanoparticles have been employed for the delivery of cancer vaccines. Additionally, oncolytic viruses (OVs), a kind of engineered virus that can selectively propagate within and destroy tumor tissue, have also been used to prime T cell responses.^[^ ^132^ ^]^

### 5.1 VLPs

VLPs are self‐assembled particles made up of virus‐derived capsid or envelope proteins to mimic the structure and function of natural viruses.^[^ ^133^ ^]^ VLPs are non‐infectious and non‐replicative since they lack the viral gene material.^[^ ^133^ ^]^ Multiple cargos can be loaded on VLPs via a variety of modification strategies including genetic modification, chemical conjugation, and non‐covalent modification.^[^ ^134^ ^]^ Some VLPs have targeting abilities due to their tropism to specific organs or cells, for example, hepatitis B VLPs and papilloma VLPs could specifically target liver and APCs, respectively.^[^ ^135^ ^]^ VLPs can also achieve LN‐targeting via the modification of targeting ligands or passive transportation by optimizing the sizes (20–200 nm).^[^ ^136^ ^]^

Although the mechanism is not fully elucidated, some studies have demonstrated the intrinsic adjuvant properties of certain VLPs for in situ vaccination and TME reprogramming.^[^ ^137^ ^]^ The first VLPs for cancer immunotherapy were reported by Lizotte et al. They constructed a 30 nm icosahedral‐structured Cowpea mosaic virus (eCPMV) VLP that constituted 60 copies of each small and large coat protein units. After eCPMV inhalation, tumor‐infiltrating neutrophils and activated neutrophils significantly increased, leading to a delayed growth of tumor and rejection to a secondary challenge.^[^ ^133^ ^]^ The immunogenicity of eCPMV was proved to be controlled by MyD88‐dependent TLR2 and TLR4 signaling.^[^ ^138^ ^]^ This neutrophil‐based antitumor strategy was successfully applied in multiple tumor models, including lung, skin, ovarian, colon, and breast cancers.^[^ ^133^ ^]^

Moreover, VLPs can serve as a vaccine scaffold to transport exogenous antigens to APCs and prime a broad but specific immune response. Li et al. loaded phage P22‐derived VLPs with B cell and T cell epitopes of OVA (OVA_B_ peptide and OVA_T_ peptide) via fusing with the C‐terminal of the coat protein. The results showed that VLP‐OVA_B_ could induce robust antibody production (titers > 10^5^), while VLP‐OVA_T_ induced effective cross‐presentation by DCs and subsequent proliferation of OVA‐specific CD8^+^ T cells. In EG.7‐OVA tumor models, three doses of VLP‐OVA_T_ together with poly(I:C) significantly delayed the tumor growth by inducing strong immune activation and immune memory, as well as reversing the immunosuppressive TME.^[^ ^139^ ^]^

### 5.2 Virosomes

A virosome is a phospholipid bilayer spherical vesicle with a mean diameter of 20–150 nm and covered with a viral envelope of glycoproteins.^[^ ^131a^ ^]^ Several viruses have been employed to generate virosomes, such as influenza virus,^[^ ^140^ ^]^ hemagglutinating virus of Japan (HVJ),^[^ ^141^ ^]^ and Sendai virus.^[^ ^142^ ^]^ The incorporation of viral envelope proteins may impact the efficiency of cargo delivery and the immunogenicity of viral particles.^[^ ^143^ ^]^ For example, hemagglutinin could bind to the cellular receptor sialic acid and undergo pH‐induced conformational changes to trigger the fusion of the virosomal and endosomal membrane, which promotes the internalization of virosomes by APCs and the subsequent cytosol transportation.^[^ ^144^ ^]^ The cytoplasmic delivery of exogenous antigens initiates the cross‐presentation process, leading to cooperation between humoral and cellular immune responses. An in vitro work showed that fusion‐competent virosomes (FCVs) were able to transport antigen OVA to DCs for MHC class I presentation at picomolar concentration, while fusion‐incompetent virosomes or FcγR‐targeted liposomes failed to generate MHC class I‐OVA complex at concentrations up to 10 nm. Notably, FCVs also showed similar efficiency in terms of MHC class II presentation, suggesting that the FCV was an efficient vector for cancer vaccine delivery.^[^ ^145^ ^]^ Besides, HVJ envelope (HVJ‐E) virosomes were reported to generate intrinsic anti‐tumor activities by activating multiple anti‐tumor immune pathways by exogenous RNA and F protein of HVJ.^[^ ^143^ ^]^ So far, due to the ease of modification and production as well as a low toxicity, two virosome‐based vaccines (Inflexal® V for influenza and Epaxal® for hepatitis A) have been successfully marketed, and another that targets melanoma was being tested in phase I/II clinical trials.^[^ ^146^ ^]^

### 5.3 Virus‐mimetic nanoparticles

Although viruses are talented at entering host cells, several limitations have hindered their clinical application, such as the small capacity for cargo‐loading, lack of targeting specificity, immunogenicity, and potential insertional mutagenesis.^[^ ^147^ ^]^ Thus, the concept of “virus‐mimetic nanoparticles,” or “artificial virus,” which refers to any bioinspired synthetic nanomaterials with virus‐like characteristics and structures, has been put forward and attracted great attention.^[^ ^147^, ^148^ ^]^

Particles of virus‐like size reach LNs efficiently, with an optimal size being ∼ 40 nm.^[^ ^149^ ^]^ Studies also have shown that antigen of particle form, particularly with a similar size to the virus, could be processed and presented by MHC class I molecules more readily than its soluble form.^[^ ^150^ ^]^ In addition, the shape of viruses is a key factor that affects their biodistribution and targeting properties.^[^ ^131b^ ^]^ The filamentous influenza virus displayed higher specific infectivity than a spherical virus.^[^ ^151^ ^]^ To imitate the structure of virus particles, Molino et al. designed a hollow structural core consisting of the E2 subunit of pyruvate dehydrogenase. A melanoma‐associated gp100 epitope and CpG were loaded on the E2 nanoparticle simultaneously, which led to a remarkable increase of antigen‐specific CD8^+^ T cells in both draining LNs and spleen. In a prophylactic B16‐F10 melanoma tumor model, a single immunization with this nanovaccine delayed tumor growth onset by ∼5.5 days and prolonged survival time by ∼40%, compared to mice treated with phosphate buffered saline.^[^ ^152^ ^]^

The multivalency of ligand‐receptor binding based on the repetitive and regular architecture of viruses endows viruses with the versatility to traverse various biological barriers.^[^ ^148^, ^149^ ^]^ A common design approach to mimicking the function of viruses is to modify artificial NPs with different types of natural viral surface elements such as attachment factors, cell‐penetrating peptides, fusion proteins, as well as antigenic peptides.^[^ ^131b^ ^]^ A better understanding of protein‐protein interactions and suitable arrangement of all required functional components in a single nanoparticle are essential but challenging.

### 5.4 OVs

OVs are therapeutics that utilize native or genetically modified viruses to selectively propagate in tumors and kill tumor cells.^[^ ^132^, ^153^ ^]^ Clinical trials have validated the therapeutic effect in cancer patients, and as a milestone in the field, talimogene laherparepvec has been approved by FDA in 2015 for the treatment of advanced melanoma.^[^ ^154^ ^]^ It is generally believed that OVs generate antitumor activity via two distinct mechanisms: direct lysis of tumor cells and induction of subsequent systemic antitumor immunity.^[^ ^153^ ^]^ OVs can cause ICD and stimulate innate immune receptors on APCs; besides, they play important roles in promoting T cell infiltration and tumor recognition, and circumventing immune suppression. All these contribute to a robust antitumor immune response.^[^ ^132^ ^]^

Since nonspecific sequestration, pre‐existing antivirus immunity, and neutralizing antibodies in the host all pose barriers to systemically administered OVs, several approaches have been developed to ensure a stealth effect in the bloodstream.^[^ ^155^ ^]^ Oncolytic Adenoviruses (OA) coated with bioengineered cell membrane nanovesicles (BCMNs) that were modified with targeting ligands achieved robust antiviral immune shielding and targeting ability. PreS1 (a ligand for sodium taurocholate cotransporting polypeptide, NTCP) was first embedded on the cell membrane by genetic engineering. In the HepG2‐NTCP subcutaneous tumor model, OAs coated with BCMNs‐preS1 (OA@BCMNs‐preS1) significantly accumulated at the tumor site. Notably, the mice treated with OA@BCMNs‐preS1 exhibited delayed tumor growth and prolonged survival time compared with the naked OA‐treated group. To further engage BCMNs as multifunctional nanoplatforms for OA delivery, a tumor‐targeting ligand was expressed on the surface of the RBC membrane via in‐body genetic engineering and coated onto OAs. The final product could conceal from the host immune recognition and specifically target cancer cells.^[^ ^155a^ ^]^

**FIGURE 6:**
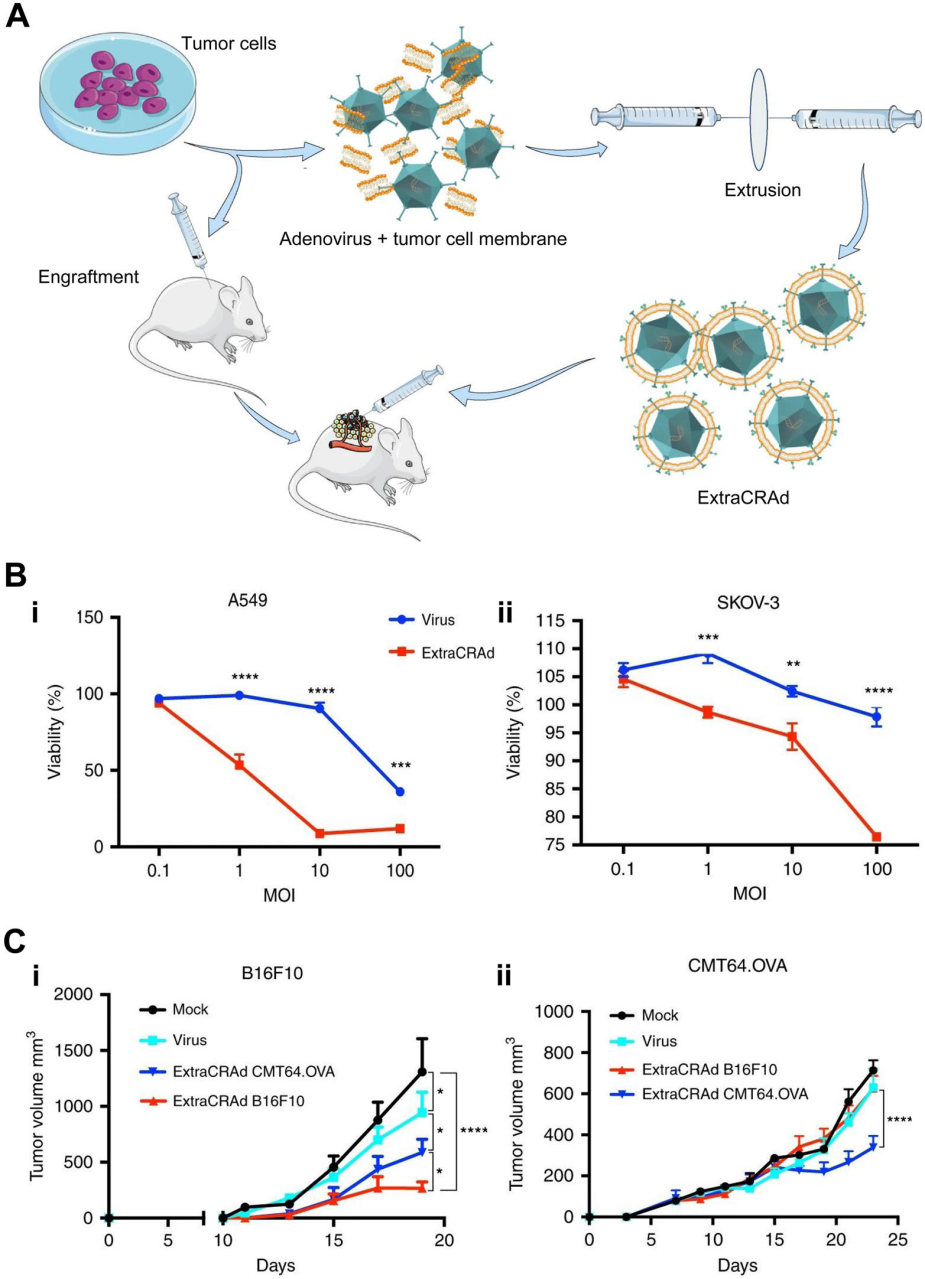
The cancer cell membrane‐coated oncolytic virus (extra conditionally replicating Adenoviruses, ExtraCRAd) for cancer immunotherapy. (A) Schematic illustration of the production and treatment of ExtraCRAd. (B) Infectivity assay of ExtraCRAd on (i) A549 (with a high level of the expression of the human Coxsackie and Adenovirus receptor, CAR) and (ii) SKOV‐3 (with a low level of the expression of CAR) cell lines. The two cell lines were infected with naked virus (blue) or ExtraCRAd (red). Cell viability was assessed by MTS assay on day 3 post infection. (C) Mean tumor growth curve (mm^3^) in a preventive setting in (i) the B16.F10 model and (ii) the CMT64.OVA model. Mock, phosphate buffered saline; virus, naked oncolytic virus; ExtraCRAd CMT64.OVA, ExtraCRAd wrapped with CMT64.OVA cancer cell membrane; ExtraCRAd B16.F10, ExtraCRAd wrapped with B16.F10 cancer cell membrane. Reproduced with permission.^[^ ^157^ ^]^ Copyright 2019, Springer Nature.

Sometimes OVs‐mediated in situ vaccination is insufficient to induce demanded T cell responses. In particular, OV‐induced antiviral responses may dominate the immune response over tumor‐specific T cell responses.^[^ ^132^ ^]^ One solution is to equip OVs with one or more exogenous TAAs.^[^ ^156^ ^]^ Fusciello et al. designed an artificially membrane‐cloaked virus via the co‐extrusion of virus and cancer cell membrane. The hybrid OVs bypassed the recognition by the classical viral receptors such as Coxsackie and Adenovirus receptors, leading to an increase in the infectivity in a viral receptor‐independent manner. Meanwhile, an increase in macrophage and DC populations were observed within the TME after the OV treatment, most of which were activated to present tumor antigens to fight against cancer. In both aggressive melanoma and lung cancer models, homologous tumor membrane‐enveloped viruses inhibited the tumor growth more significantly compared with allogeneic ones or naked viruses, which indicated the importance of appropriate equipment of tumor‐specific antigens on the OVs (Figure 6).^[^ ^157^ ^]^

## 6 CONCLUSIONS AND OUTLOOK

Harnessing the immune system to attack the tumor by cancer vaccines is being intensively studied. The unique properties of the natural biological system offer exceptional clues for the design of cancer vaccines to address specific needs. For example, being the only FDA‐approved cancer vaccine, the DC‐based sipuleucel‐T vaccine could be potentially replaced by artificial APCs as a functional‐competent alternative to reducing the time‐consuming and costly procedure of live cell manufacturing. A successful vaccination usually requires the coadministration of adjuvants. By taking advantage of the natural products, a large number of self‐adjuvanted biomaterials could be integrated for constructing effective vaccines. Furthermore, bacteria‐ or virus‐derived components such as OMVs and VLPs that retain the immunostimulatory components can sensitize the tumors without introducing other therapeutic agents, serving as potent in situ vaccines against cold tumors.

The success of antigen‐specific cancer vaccines relies heavily on the optimal choice of antigens.^[^ ^5^, ^158^ ^]^ TAAs, referring to those that are overexpressed in cancer cells but modestly expressed in normal tissues, were the popular candidates for vaccine development for many years; however, TAAs may be subject to central and peripheral tolerance and lack complete tumor specificity.^[^ ^158^ ^]^ Recently, neoantigens seem to become more attractive for the development of personalized cancer vaccines, as they are generated via somatic mutations exclusively existing in individual patient's tumors but not normal tissues.^[^ ^159^ ^]^ Neoantigens are not subject to immune tolerance compared to TAA. In addition, patient‐specific neoantigens (typically composed of a pool of predicted antigens/epitopes) could induce a more diversified T cell response against tumor compared to a single TSA. Therefore, neoantigen vaccine may generate a long‐lasting and broader T cell immunity against the heterogenic primary tumor lesion and tumor metastases.^[^ ^160^ ^]^ Although being an intriguing approach, patients who are willing to receive neoantigen vaccine may miss the optimal treatment window due to the long sequencing and preparation cycle (3–5 months).^[^ ^161^ ^]^ To accelerate the producing process, an alternative consideration is to develop neoantigen vaccines using “public neoantigens” derived from hotspot mutations in driver oncogenes.^[^ ^162^ ^]^ Regarding the treatment of tumor with a low mutation burden, TSA or TAA vaccine may be more feasible. Patient‐specific tumor cell‐inspired cancer vaccines is another attractive alternative as they retain both TAA and TSA of patient‐specific tumor which could meet the increasing demand for personalized therapy with readily available biomaterials.

While successes have been shown in cancer vaccines inspired by eukaryotic cells, bacteria, or viruses in preclinical and clinical studies, not a single biomimetic or bioinspired nanovaccine has entered the market. Although preclinical data suggest the trending of application of nanotechnology in immunotherapy,^[^ ^163^ ^]^ several concerns and issues still need to be addressed to pave the road of nanovaccine to the clinic. First, the common issues that existed for all cancer vaccines also apply to biomimetic and bioinspired nanovaccines including the lack of protocols for Good Manufacturing Practice, a scale‐up manufacturing process for producing stable, and sterile products with constant quality. For example, distinct to the synthetic material, biomembrane‐based nanostructures, such as exosomes and OMVs, suffer from the time‐consuming and low‐efficient production process. Being the most widely used isolation technique, the ultrafiltration method is limited by membrane clogging and vesicle trapping during exosome preparation.^[^ ^164^ ^]^ Several recent reviews have summarized the current production improvement approaches.^[^ ^165^ ^]^ Microfluidics provides integrated platforms and demonstrates fascinating separation and sensing capabilities for exosome isolation in combination with conventional techniques.^[^ ^166^ ^]^ Genetic modification or culture medium optimization of donor cells/bacteria was also applied to improve the production and stability of membrane vesicles such as exosomes and OMVs.^[^ ^165^ ^]^ Second, in specific, to yield a constant production for biomimetic nanovacines, the synchronization and quality control of the parental cells need particular attention. The heterogenetic cell properties make the products somehow unmanageable and vary across batches. Some synthetic materials, such as lipids and polymers, have demonstrated the advantage of their versatility in cancer vaccine delivery.^[^ ^167^ ^]^ Hybridization of the synthetic materials with natural structures will potentially generate a more defined composition and controllable functionality for vaccination. For example, lipid molecules can be readily inserted into natural cell membranes to serve as the anchor for modified motifs or execute their inherent functions (i.e., stimuli‐responsive).^[^ ^168^ ^]^ An alternative avenue is to fuse natural cell membranes with pre‐engineered liposomes to construct hybridized vesicles with both natural and engineered features.^[^ ^169^ ^]^ Third, to expand the potential application of vaccine in clinical settings, a combinational approach may be required. Cancer vaccines administered as monotherapy are modestly effective owing to the T cell resistance mechanisms within the TME.^[^ ^6^ ^]^ Therefore, the combination of cancer vaccines with other modalities such as immune checkpoint inhibitors becomes more feasible. The former could turn the immune cold tumors into hot tumors, while the latter reverses the immunosuppressive TME. A clinical attempt succeeded in a phase II clinical trial with a double of overall response rate and survival.^[^ ^170^ ^]^ The combination of cancer vaccines and CAR‐T immunotherapy has also been reported, where vaccines loading with CAR antigen drove the expansion of CAR‐T cells against solid tumors.^[^ ^45^, ^171^ ^]^ Last, although an appropriate level of immunogenicity is attractive for cancer vaccines, the assessment of safety is still of great importance in clinical translation. For eukaryotic cell‐inspired cancer vaccines, the use of allogenic cells reduces the financial burden and the waiting time compared to an autologous source but may generate the risk of immune rejection due to MHC mismatch. Gene‐editing techniques may greatly enhance the compatibility of allogeneic cells. The establishment of genetic modified allogeneic cell bank or induced pluripotent stem cell bank is suggested to facilitate the access to appropriate donor cell sources for clinical use. For bacteria‐inspired cancer vaccines, the introduction of endotoxins needs to be carefully assessed prior to the clinical application since the safety issues of bacteria derivatives have been widely reported.^[^ ^116^ ^]^ The toxic molecules could be downregulated or knocked out with the assistance of genetic engineering. The development of novel isolation and purification techniques is also one of the directions to improve safety. The profiling of the natural components remains to be fully characterized especially the molecular composition and metabolites through proteomics and metabolomics analysis. The above information is necessary for the understanding of the immunomodulation mechanism, the assessment of safety, and the establishment of regulatory guidelines for production.

## CONFLICT OF INTEREST STATEMENT

The authors declare no conflicts of interest.
